# Home-based palliative care management: what are the useful resources for general practitioners? a qualitative study among GPs in France

**DOI:** 10.1186/s12875-020-01295-7

**Published:** 2020-10-31

**Authors:** Carol-Anne Boudy, Tiphanie Bouchez, Didier Caprini, Isabelle Pourrat, Stéphane Munck, Adriaan Barbaroux

**Affiliations:** 1 Université Cote d’Azur, Rétines, DERMG, Nice, France; 2 Université Cote d’Azur, Rétines, LAPCOS, DERMG, Nice, France

**Keywords:** Palliative care, Family practice, Qualitative research, Primary care

## Abstract

**Background:**

Most French people (71%) would like to die at home, but only one out of four actually do. While the difficulties inherent in the practice of home-based palliative care are well described, few studies highlight the resources currently used by general practitioners (GPs) in real life. We have therefore sought to highlight the resources actually used by GPs providing home-based palliative care.

**Methods:**

Twenty-one GPs of different ages and practice patterns agreed to participate to this qualitative study based upon semi-structured interviews. They were recruited according to a purposive sampling. Transcripts analysis was based upon General Inductive Analysis.

**Results:**

The resources highlighted have been classified into two main categories according to whether they were internal or external to the GPs. The internal resources raised included the doctor’s practical experience and continuous medical education, personal history, work time organization and a tacit moral contract related to the referring GP’s position. External resources included resource personnel, regional assistance platforms and health facilities, legislation.

**Conclusion:**

This study provides a simple list that is easy to share and pragmatic solutions for GPs and policymakers. Home-based palliative care practice can simultaneously be burdensome and yet a fulfilling, meaningful activity, depending on self-efficacy and professional exhaustion (burnout), perhaps to a greater extent than on medical knowledge. Home-based palliative care promotion is a matter of social responsibility. The availability of multidisciplinary teams such as regional assistance platforms and Hospitalization at Home is particularly important for the management of palliative care. Policymakers should consolidate these specific resources out of hospitals, in community settings where the patients wish to end their life.

**Supplementary Information:**

The online version contains supplementary material available at 10.1186/s12875-020-01295-7.

## What was known

- The main difficulties towards fulfilling home-based palliative care are a feeling of isolation, a lack of time and training, the emotional suffering endured by healthcare workers and communication issues between GPs and hospitals.
- Despite a relative lack of specific training, GPs do use a patient centeredness approach and work as a multidisciplinary team to anticipate events, while simultaneously involving the patient’s family in the medical decisions.

## What the article brings

- The moral responsibility related to a long-time relationship between a GP and a patient was seen as a resource more than a burden.
- Collaboration with multidisciplinary structures, from primary or secondary care, was a key resource.
- Personal characteristics such as vision of healthcare’s relationship, burn-out status and self-efficacy were more involved in how GPs experienced end-of-life management than knowledge or education path.

## Background

The French National Palliative Care Development Program defines access to homecare as a National Priority [1]. In France, general practitioners (GPs) have in average 850 persons on the patient list and therefore follows ten end-of-life patients a year. One to three of them will die at home each year [2]. National home-based death rate is 24.1%, with low inter-region variability [3]. In the Provence-Alpes-Côte d’Azur (PACA) region, the home-based death rate is 27%, which is among the highest rates in France.

According to a report published by the End-Of-Life National Observatory, 75% of the French population’s deaths occur in hospitals or nursing care facilities. Yet, 81% of the French population wishes to die at home, and only 10% changed their mind after being confronted with the end of their life [2, 4]. According to this report, the way home-based end-of-life care is conducted in France has to improve. At the European level, palliative care is often summed up as relieving physical symptoms for the patient in the terminal stage [5]. Practitioners are not often aware of their patients’ wishes with respect to the end of their life. Yet, when the practitioners are informed, the patients more often die where they prefer [6].

A systematic review from Australia [7] showed that practitioners consider palliative care to be a gratifying part of their profession and that patients do appreciate their involvement. In the Netherlands, a qualitative study showed that GPs describe palliative care as a fulfilling task which can sometimes become a burden [8]. The main disincentive seems to be the emotional burden that leads to healthcare workers distress and sometimes leads professionals to disengage [1]. Such distress is worsened by a feeling of isolation, a lack of time, a lack of training, a hardships associated with the patient’s relatives, problematic access to certain drugs and a lack of communication between GPs and hospitals [2].

A 2014 French study studied the experiences of the practitioners and the skills involved during home-based palliative care, with the goal of identifying their needs. Despite a relative lack of specific training, the practitioners used a patient-centeredness strategy and worked as multidisciplinary teams to plan ahead, while involving the patient’s family in the medical decisions [9]. This study was centered around practitioner experiences and their training needs, but did not address the resources that these practitioners used.

All in all, numerous studies have investigated the circumstances that prevent home-based palliative care, but very few of them describe which resources are used by the practitioners who perform home-based palliative care. The goal of this work is to identify the resources used by GPs for home-based palliative care in France.

## Methods

### Design

A qualitative study was conducted through semi-structured interviews. The analysis was based upon General Inductive Analysis [10, 11]. The research protocol and the presentation of results are based upon COREQ criteria (COnsolitaded criteria for REporting Qualitative research; 12). COREQ is a 32-item checklist that can help researchers to report important aspects of the research team, study methods, context of the study, findings, analysis and interpretations [12]. The use of COREQ is recommended by the EQUATOR network (Enhancing the QUAlity and Transparency Of health Research [13]);.

### Participant characteristics

The interviewed doctors practiced in both rural and urban areas of the Alpes-Maritimes. No non-inclusive criteria were set. A purposive sampling was used to obtain a diversity of GP experiences across various characteristics such as age, sex, work setting (alone or in a team, urban or rural), and years of practice. The purposive sampling technique is the deliberate choice of a participant due to the qualities the participant possesses. Simply put, the researcher decides what needs to be known and sets out to find people who can and are willing to provide the information by virtue of knowledge or experience [14]. Practitioners were identified from a phonebook and recruited through phone calls. An oral principle agreement was established during the first phone call.

### Data collection

An interview guide (see additional file 1: interview guide) composed of open-ended questions was developed by the research team. It evolved to adapt to the exploratory approach according to the analysis of recent data as the collection progressed. Interviews were conducted on a face to face basis, at the practitioner’s office. They were structured in a conversational mode without any note taking, so as to create an atmosphere of trust and to favor free speech. The interviews were recorded with an audio recorder and then transcribed word for word. The main investigator (CAB) presented herself as a general practice intern and described the study as her thesis work.

### Data analysis

The analysis method was based upon General Inductive Analysis. General Inductive Analysis seeks to distinguish the most indicative theme categories rather than form a theory based on those newly identified themes [10]. This method highlights hypotheses and thus corresponds to the exploratory goal of the research [11, 15].

Data analysis was done in teams, including three GPs with experience in qualitative research (IP, DC, AB). Data were transcribed and analyzed in Microsoft Word© and didn’t use any specific qualitative data analysis program.

An intermediate analysis was performed every three interviews until reaching data saturation, hereby defined as analyzing three subsequent interviews that did not offer any new concepts. The analysis and encoding of the transcriptions for each interview were controlled by two different persons among AB, DC, and IP, so as to minimize any interpretation bias (data triangulation). Any disagreement was discussed until a consensus was reached between the researchers.

### Reliability criteria

General Inductive Analysis enables a precise analysis of the transcriptions and limits any interpretation bias related to the investigator. This stems from the assumption that the qualitative approach is coherent with our exploratory goal. The main investigator (CAB) obtained preliminary training for semi-structured interviewing by reading reference books and via the Qualitative Research Methodological Department of the Medicine Faculty. The intermediary analysis systematically involved the main investigator and two practitioners with expertise in qualitative research (IP and DC).

### Ethical considerations

The study was conducted according to French regulations at the date of data collection. Participants provided an oral agreement, and they were informed they could stop the interview at any time without having to justify their decision. The transcriptions were anonymized and the interviews were identified with a number. The authors chose not to identify the interview number after each quote to prevent any possibility of identification by cross-referencing data.

## Results

### Population characteristics

Twenty-one interviews were made from August 2014 to February 2015 and required 113 phone calls. Their average length was 25 min and varied from 14 to 57 min. Data saturation was reached after 18 interviews. During the interviews, two practitioners declared that they did not perform home-based palliative care. The researchers analyzed the related transcriptions anyway, but they did not offer any novel data on the research topic. The characteristics of each interviewed practitioner are described in the supplementary materials. The GPs had been practicing for 6 to 40 years. Most of them (eighteen) were established in urban areas.

The interview guide was only slightly modified over the course of the study. The first two questions were reworded: “When you managed your first palliative care patient, what was the main problem you had to face?” was replaced with “When you first set up, what were the main problems you were confronted with when managing palliative care patients?”. The question “And as for your most recent palliative care patient? How did your management differ?” was replaced with “And today, does it seem simpler? What solutions did you find?”. These modifications aimed to encourage broader thinking, to help the interview subjects to remember past events, and to focus the discussion on solutions rather than problems.

### General inductive analysis

The analysis helped highlight 10 main categories which can be sorted into two groups: resources that are *internal* to the practitioner and resources which are *external*. *Internal resources* are hereby defined as the set of resources directly related to each practitioner: practical experience and continuous medical education, personal history, work time organization and a tacit moral contract linked with the referring GP’s position. *External resources* are hereby defined as the resources related to the territory: resource personnel, regional assistance platforms and health facilities, legislation.

### Internal resources

#### Practical experience and continuous medical education

The practitioners described their practical experience in managing home-based palliative care as a resource simultaneously allowing the acquisition of theoretical knowledge and creating a professional network. That knowledge was expanded through communication with peers, experiential knowledge and the continuing medical education. *“I realized that I knew much less than doctors who only do palliative care, but I learned to compensate incredibly with my experiential knowledge, it’s amazing!”.* No practitioner spontaneously broached initial medical training as a resource for palliative care. The interview guide planned to broach the topic, but most practitioners considered that their initial theoretical training was of little help to them. However, internships were often quoted as a resource for home-based palliative care. Internship is mandatory in France during 3 years before to settle. Internships were helpful to home-based palliative care when they permitted GPs to learn how to give patients the news of a serious illness, to practice palliative care and to face death. GPs described internships as a way to gain experience and self-confidence.

#### Personal history

Personal history, family and privacy were described as resources that were relied upon by the practitioners. Facing death or home-based palliative care within their own families helped them face the moral difficulties inherent to home-based palliative care. “*I talk about it with my wife, she knows a bit about it”;* “*I run (laughs), I do a lot of running and it helps me*”; “*in the end, we also experience that for ourselves, our family, our parents, and so on.*”. Some practitioners described their capacity to stand back from death. Standing back helped them to face moral hazard inherent to home-based palliative care. “*I have patients that I’ve been taking care of for 20, 30, 35 years, and they are people [ …] for whom I know that at a given time, they will have to go [ …] I learned, and I experienced.*”

#### Work time organization

Some practitioners included emergency time windows in their work planning and programmed frequent visits for people in palliative care. This organization of work time, according to them, enabled efficient management of the time-consuming aspect of home-based palliative care and helped to anticipate complications and to recreate the balance between the time dedicated and financial gains.

#### Referring GP’s position

In France, the referring GP plays a central role in the organization of home-based palliative care and Hospitalization at Home. “Referring GP” corresponds to an administrative function of GPs in France: patients have to officially choose their referring GP. The refund of medical care treatments depends on the declaration of this choice to Health Services and on the respect of the coordinated care process, in which one must seek medical attention from one’s referring GP as the very first step. This administrative formality leads to a tacit involvement from the GP. This involvement was considered to be both an asset and a problem for the interviewed GPs. Indeed, the referring physician’s status sometimes represented a heavy commitment. However, they knew how to rely on this tacit moral contract to give added value to their management. GPs from our sample described this sensitive status as a catalyst for shared medical decisions and a patient-centered approach. According to them, this approach aimed at breaking the isolation when facing difficult decisions and encouraged a quality relationship between a practitioner and their patient. One practitioner declared that shared decision making brought value to his work and reduced his concerns about legal risks. GPs also outlined how important positive and gratifying feedback from the patient and their relatives is. The “*feeling of being useful*” was described as a resource that helped them face the most difficult moments.

The status of a “family doctor” appeared to help GPs cope with the workload and the time spent at the patient’s side. “*That’s the humanistic aspect of our trade, that’s the beautiful aspect*”. GPs did outline how important a quality GP/patient relationship is; as well as knowing the patient, their relatives and their social/family conditions. The quality of such a relationship also seemed to help them cope with the workload and the time spent at the patient’s side through a tacit moral contract. This tacit moral contract sometimes led to a care obligation: “*It is a family, and we do take care of our medical family.*” “*That’s my philosophy to an extent, I’ve treated these people and they allowed me to pay my bills for 30 years, so granting them a bit of time at the end of their life isn’t an issue.*”

Several practitioners considered that providing palliative care consisted of placing their moral contract and their commitment to the patient ahead of their comfort in daily practice. During difficult moments, they relied on their moral values as a resource: “*It’s an honor! They believe in me and I cannot give up on them.*” This consideration seemed to alleviate the feeling of burden and helped them keep a sense of proportion in the problems they encountered when practicing home-based palliative care. The difficulties incident to home-based palliative care were compensated by a feeling of moral gratification: “*Sometimes it’s difficult, but in times like these, you feel like you’re really needed.*”

### External resources

#### Resource personnel

All the GPs of the sample did evoke how important it is to rely on resource personnel. The most cited resource personnel were the freelance nurses with whom the practitioners did build trust relationships allowing them to break the isolation. The practitioners did also appreciate the possibility to delegate certain tasks to them, such as managing patients’ anguish. The other relevant resource personnel were medical or paramedical, liberal, or hospital professionals, as well as other network members. Informal contact between medical professionals was described as vital. For example, one of the practitioners regularly called the regional palliative care assistance platform’s doctor to get advice even for patients who were not managed by this platform.

The patients’ relatives were often considered as a resource during decision making. Including the relatives in the shared medical decision process helped to lift the weight in some decisions. Furthermore, the patient’s relatives brought logistical support and helped to manage treatments.

#### Regional assistance platforms and health facilities

Hospitalization at Home (HAH), palliative care mobile teams and medical networks were described as almost indispensable structures to the outpatient practice of palliative care. HAH was acclaimed and considered as a weight lift in the workload. “*HAH structures provide good oversight, there are regular visits, and we are informed at the slightest problem.*” The interviewed practitioners declared that they used the palliative care mobile teams for logistical support but also for training support, prescription assistance, and help in assessing the situation and needs of the patient. Regional assistance networks brought the same benefits without replacing the usual private practitioners with the ones from the structure. The GPs did appreciate the concept of retaining the power of decision. Finally, the Palliative Care Units and the other hospitalization services were described as a resource in the practice of home-based palliative care while allowing some temporary relief for the care staff.

#### External resources to fight against isolation

GPs described mechanisms to fight against isolation. They would use the structures, the resource personnel, shared medical decision making (with the patient but also with their relatives), the writing of advance care directives, continuous medical training, peer groups and communication with hospital services to successfully cope with the hazard of feeling isolation.

#### Legislation

Several practitioners evoked a fear of having to face legal repercussions. This fear could even lead them to make decisions which would be contrary to the patients’ interests. “*From now on, our own interest must be a priority, ahead of the patients’. […] There are only two medical magazines to read:* [prescriptions]© *and* [liability]©.” French legislation on palliative care (“*Leonetti law”*) was however described as a factor that would facilitate the management of palliative care because it gives to palliative care and Active Therapeutic Acts Limitation “*more legitimacy*.” This law was also described as a support to help differentiate between euthanasia (illegal in France) and palliative care and thus manage the patient’s and/or the family’s requests.

## Discussion

This study highlights the applicable resources that are used by GPs in France during the management of patients in home-based palliative care. These include logistical resources such as dedicated infrastructure (regional assistance networks or home-based hospitalization), as well as *internal resources* that are specific to each practitioner (a tacit moral contract, time management, training, experiencing bereavement). This study does not provide an exhaustive list, but describes the resources actually used by the practitioners in a region that can be considered to be a model because of its rate of death at home, which is one of the highest in France.

### Home-based hospitalization: a solution against isolation?

Isolation is a problem often evoked when talking about palliative care, but also more widely regarding liberal medicine [16, 17]. In our sample, regional assistance networks and home-based hospitalization were described as an efficient solution to social isolation. These findings are consistent with data from previous studies [9, 18–22]. In our study, a multidisciplinary approach such as Hospitalization at Home was deemed necessary for the success of outpatient management when reaching the end of their life. This result match data from previous studies [23–25] which show that Hospitalization at Home improves care quality, reduces the number of re-hospitalizations, and is satisfying to patients. Unfortunately, its availability is very heterogeneous according to the area: many French towns do not have such access. This inequality in accessing palliative care can also be found on an international level: home-based hospitalization structures can be found in Canada, USA and Switzerland, whereas Italy or Belgium only have much smaller home-based care structures [26].

The participants considered that sharing the medical decision with the patient and their relatives encouraged a quality relationship between the patient and their physician. These results are consistent with the work of Oude Engberink et al. who emphasized the necessity for patient centeredness in home-based palliative care [9]. According to the End-Of-Life National Observatory Report, [2] the process of choosing where the patient wants to die is more important than the final decision, and the quality of such a choice depends on the respect for the patients’ values as well as the availability of adequate information and the patient’s involvement in making the choice: this is the principle of shared medical decisions [27–32]. This concept is closely related to that of Evidence-Based Medicine as described by Sackett [33]. When associated with the communication between a patient and their physician, this model improves the acceptance of treatment, the patient’s satisfaction and the practitioner’s satisfaction [28–34]. Hence, the link between the referring physician and shared decision making seems particularly important for home-based palliative care.

### Differential experiences

In spite of similar work conditions, some GPs felt supported and helped while others felt alone with the prospect of a difficult task. Beyond the question “*what are the existing resources?*” this observation reveals differences in how the GPs felt towards available resources.

According to the sample described here, authors suggests that this observation can be explained by self-efficacy, the theory of planned behavior and by the medical professional exhaustion syndrome. Indeed, some internal resources outlined by this study can be regrouped within Bandura’s self-efficacy concept [35]. This concept can be defined as the belief that one has in one’s own capacity to perform a task or not. The theory of planned behavior states that behavioral intentions and behaviors are shaped by intention toward behavior, subjective norms, and perceived behavioral control (self-efficacy). The feeling of self-efficacy underpins the motivation required in order to perform an action or not. Hence, the problems inherent to palliative care practice can become more or less powerful obstacles depending on the GPs’ character and their self-efficacy. According to the theory of planned behavior, [36] intention to practice home-based palliative care depends on both knowledge, skills and self-efficacy.

The medical professional exhaustion syndrome (burnout) is a frequent pathology [37]. It can obstruct the palliative care performed by GPs by notably leading to a disinvestment in the professional activity, and to a feeling of failure and incompetence [38]. The difficulties for the referring physician to switch from a “life viewpoint” lasting several years, to an “end-of-life viewpoint” do worsen the GPs’ suffering and expose them to burnout [39]. The disinvestment of some GPs can thus be related to a defense mechanism [18–20, 38, 40].

### The place of the law

French laws related to the end of life have been heavily punctuated with numerous changes over the last 20 years. In 2002, the “Kouchner” law states that any sick person who requires it can access palliative care and support and that any ailing person can oppose any testing or therapy. The lawmakers specify that anybody can receive care to relieve pain. The latter is, in any circumstance, prevented, assessed, taken into account and cared for. In 2005, the “Leonetti” law initiated the writing of advance care directives concerning the limitation or cease of any type of treatment (including feeding or hydration) and the sedation during the terminal stage. In 2016, the Claeys-Leonetti law stated that “*anybody has the right to a dignified and peaceful end of life. Health professionals implement every available means for this right to be respected.*” [41] Some practitioners in our study felt the Leonetti Law prevented home-based palliative care while others depicted this law as a resource that was helping them. Such discordance can be explained by a lack of legal knowledge and by a reluctance linked to a perceived loss of freedom [8].

### The place of training

The limited training level of GPs can be seen in other studies [40, 42–45]. The difference in the use and knowledge of palliative care medical network structures has also been previously described [20, 46–49]. Palliative Care Inter-University Diploma (*Diplôme Inter-Universitaire*) is a marginally used resource: only 2.6% of French GPs benefited from it between 2005 and 2009 [4]. Interviewed GPs did not consider they required additional training and did not considered continuing medical education as a resource. This data suggests that implementing more and more knowledge-based medical education would not necessarily be deemed beneficial in that field where time is especially precious [50]. At first sight, this finding seems contradictory with existing data. Indeed, Thoonsen et al. [6, 51, 52] have shown that training GPs makes them more easily able to discuss the future with their palliative patients, to identify more palliative patients and to provide multidimensional palliative care more often. Actually, these data are complementary since the training studied by Thoonsen et al. aimed not only at providing knowledge but also skills and self-efficacy. Hence, our data and that on Thoonsen converge with the theory of planned behavior to show that to be effective, training must focus not only on the provision of knowledge, but also and above all on the provision of skills and self-efficacy.

### Strengths and limitations

Our sample was composed of 15 men for 6 women and only two GPs practicing since less than 20 years. However, different ages, genders, settlement patterns and experiences were included. Hence, this sample can be considered as diversified and matches the chosen methodological criteria [10]. Moreover, the distribution corresponds to the medical demography of the region [53, 54].

One can imagine that some GPs might not have dared confess indifference or even disinvestment in palliative care (social desirability bias). However, we believe that our interview techniques allowed GPs to speak freely. For example, two GPs readily admitted they were trying to offload palliative care. To keep it simple for informants, interviews transcripts were not sent back for comments and corrections. Data were collected 5 years ago but the context remains very similar: to date, available external resources, law and internal resources held for palliative care are very likely to remains the same.

The strengths of this work lie in the simultaneous exploration of the *internal* and *external* resources that were used by GPs in daily practice. To the best of our knowledge, our study is the first to ever ask the question of the resources used by GPs who practice home-based palliative care instead of focusing on their experience or the disincentives for palliative care practice. The interview guide was open and directed as little as possible so as not to lead the debate or introduce a subjectivity bias. The analysis bias (subjectivity of encoding and data analysis by a beginner investigator) was controlled by triangulation of data analysis. From a methodological point of view, the key strengths of this study are the data triangulation and the focus of the interviews on resources and solutions instead of difficulties, which provides relevant data for daily practice.

### Practical consequences and perspectives

Policy makers want to promote home-based palliative care to answer population needs, whereas some overwhelmed GPs are tempted to give up. This study provides a list that is simple to share and offers pragmatic solutions for GPs and for policymakers. Moreover, our findings highlight that home-based palliative care practice can be both burdensome and yet a fulfilling, meaningful activity. This balance could depend on self-efficacy and professional exhaustion, perhaps to a greater extent than medical education. Hence, palliative care support could be included in the social responsibility expectations of universities [55] but also of Regional Health Agencies, which have to introduce changes in order for palliative care practice to be known not for its constraints but for its fulfilling aspect. Such action could start right at the beginning of medical training, by presenting the fulfilling aspect of palliative care practice to students. Public campaigns focusing on the GPs’ pivotal role and financial incentives for palliative care practice could also be considered. This need for recognition of palliative care activities has also be found in other studies [19, 42] and a financial incentive has been already implemented in France for nurses and physiotherapists [56, 57].

## Conclusion

The problems related to the homebased practice of palliative care by GPs are well known, yet few studies highlight the resources actually used by GPs. This study outlines such resources, including structures (regional assistance networks, Hospitalization at Home), the use of resource personnel (medical, paramedical and family), and schedule organization.

A GP’s status implies a tacit moral contract that involves managing home-based palliative care when it is needed. This moral responsibility is more a resource than it is a burden for the GPs who rely on their care values in order to face difficulties.

Some important divergences in opinion and resource use were found. These divergences could be due to the relationship between GPs and their patient, to the burnout syndrome and to differences in self-efficacy.

In this context, the implementation of multidisciplinary teams such as regional assistance platforms and HAH structures is particularly important for the success of home-based palliative care practice for patients reaching the end of their life. Home-based palliative care is complex but fulfilling, and deserves to be recognized by policymakers in accordance with social responsibility, and to the benefit of caregivers, but above all of populations.

## Supplementary Information

**Additional file 1:.** Interview guide.

## Data Availability

The relevant datasets are available from the corresponding author Adriaan BARBAROUX (abarbaroux@unice.fr) on reasonable request.

## Abbreviations

HAH: Hospitalization at home
GPs: General Practitioners

## References

[1] *Plan National 2015–2018 pour le développement des soins palliatifs et l’accompagnement en fin de vie*. [cited 25 august 2018]. Available from: http://solidarites-sante.gouv.fr/IMG/pdf/031215_-_plabe56.pdf.

[2] Observatoire National de la Fin de Vie (2012). Fin de vie: *Fin de vie au domicile: vivre la fin de vie chez soi*.

[3] *Les décès en 2016 - Comparaisons régionales et départementales − Les décès en 2016 | Insee* [Internet]. [cited 19 juill 2018]. Available from: https://www.insee.fr/fr/statistiques/3053199?sommaire=3053204.

[4] Observatoire National de la Fin de Vie (2011). *Fin de vie: un premier état des lieux*.

[5] Meeussen K, Van den Block L, Bossuyt N, Bilsen J, Echteld M, Van Casteren V (2009). GPs’ awareness of patients’ preference for place of death. Br J Gen Pract J R Coll Gen Pract sept.

[6] Thoonsen B, Vissers K, Verhagen S, Prins J, Bor H, van Weel C (2015). Training general practitioners in early identification and anticipatory palliative care planning: a randomized controlled trial. BMC Fam Pract.

[7] Mitchell GK. How well do general practitioners deliver palliative care? A Systematic Review Palliat Med nov 2002;16(6):457–464. 10.1191/0269216302pm573oa 12465692

[8] Groot MM, Vernooij-Dassen MJFJ, Crul BJP, Grol RPTM (2005). General practitioners (GPs) and palliative care: perceived tasks and barriers in daily practice. Palliat Med mars.

[9] Oude Engberink A, Badin M, Serayet P, Pavageau S, Lucas F, Bourrel G (2017). Patient-centeredness to anticipate and organize an end-of-life project for patients receiving at-home palliative care: a phenomenological study. BMC Fam Pract.

[10] Thomas DR. A General Inductive Approach for Analyzing Qualitative Evaluation Data. Am J Eval. 27(2):237–46.

[11] Strauss A, Corbin J (2003). L’analyse de données selon la grounded theory. Procédures de codage et critères d’évaluation. L’enquête de terrain.

[12] Tong A, Sainsbury P, Craig J (2007). Consolidated criteria for reporting qualitative research (COREQ): a 32-item checklist for interviews and focus groups. Int J Qual Health Care J Int Soc Qual Health Care déc.

[13] Consolidated criteria for reporting qualitative research (COREQ): a 32-item checklist for interviews and focus groups | The EQUATOR Network [Internet]. [cited 2020 Oct 4]. Available from: https://www.equator-network.org/reporting-guidelines/coreq/. 10.1093/intqhc/mzm042 17872937

[14] Bernard HR (2002). Research methods in anthropology: qualitative and quantitative approaches.

[15] Guillemette F (2006). L’approche de la grounded theory; pour innover. Recherches qualitatives.

[16] Barbaroux A, Pourrat I, Feroni I, Bouchez T (2020). GPs and Pharma Reps: Why Are We So Ambivalent? Crossing perspectives from sociology, psychology and general practice toward a qualitative study in France.

[17] Mousquès J (2015). L’impact de l’exercice regroupé pluriprofessionnel sur la qualité des pratiques des médecins généralistes.

[18] Astier C. Quels sont les besoins et les attentes des médecins généralistes dans l’aide à la mise en place de soins palliatifs à domicile? Méd Hum Pathol. 2013; <dumas-00913148>.

[19] Sansoucy E. Facteurs limitant l’accompagnement des patients en fin de vie en médecine générale: vécu des aidants naturels. Méd Hum Pathol. 2013; <dumas-00881559>.

[20] Bec M (2020). Deep and continuous sedation maintained until death at home: general practitioners’ experiences. Exercer.

[21] Ladevèze M, Levasseur G (2010). Le médecin généraliste et la mort de ses patients. Prat Organ Soins.

[22] Cueille V (2017). Le médecin généraliste face à la fin de vie à domicile: connaissances, compétences et limites. État des lieux auprès des médecins généralistes de la Seine Maritime et de l’Eure*.* [Thèse d’exercice, médecine]. Rouen.

[23] Svensson G, Wåhlin I (2018). PatientPerceptions of Specialised Hospital-Based Palliative Home Care: a Qualitative Study Using a Phenomenographical Approach. Int J Palliat Nurs.

[24] Melsom H, Wist E (2001). Terminal care of cancer patients. Tidsskr Nor Laegeforen.

[25] Hébert S, Gravou-Apostolatou C, Rascher W (2017). Establishment of Specialized Pediatric Palliative Home Care in the Medical Valley of the European Metropolitan Area Nuremberg. Klin Padiatr.

[26] Guinet A. *Organisation des soins à domicile en Europe et en Amérique du Nord*. In: MOSIM 2014, 10ème Conférence Francophone de Modélisation, Optimisation et Simulation. Nancy, France; 2014. [cited 27 feb 2020]. Available from: https://hal.archives-ouvertes.fr/hal-01166613.

[27] Gisquet E (2006). Vers une réelle ingérence des profanes? Le mythe de la décision médicale partagée à travers le cas des décisions d’arrêt de vie en réanimation néonatale. Recherch Fam.

[28] Fournier C, Kerzanet S (2007). Communication médecin-malade et éducation du patient, des notions à rapprocher: apports croisés de la littérature, Summary. Santé Publique.

[29] La Santé de l’homme n° 383 - *A quoi sert l’éducation pour la santé pour pratiquer l’éducation du patient?* [Internet]. [cited 23 july 2018]. Available from: http://inpes.santepubliquefrance.fr/SLH/articles/383/06.htm.

[30] Llorca G (2006). L’accord mutuel librement consenti dans la décision médicale. Médecine..

[31] Pierron J-P (2007). Une nouvelle figure du patient? Les transformations contemporaines de la relation de soins. Sciences sociales et santé.

[32] Gauthier G (2015). Fin de vie à domicile et préférence pour un lieu de décès: revue de la littérature. Exercer.

[33] Sackett DL, Rosenberg WM, Gray JA, Haynes RB, Richardson WS (1996). Evidence Based Medicine: What It Is And What It Isn’t. BMJ.

[34] Taïeb S, Vennin P, Carpentier P (2006). EBM et choix du patient (no 3): avec quelle information?. Médecine..

[35] Bandura A (1977). Self-efficacy: Toward a Unifying Theory of Behavioral Change. Psychol Rev.

[36] Ajzen I, Kuhl J, Beckmann J (1985). From intentions to actions: a theory of planned behavior. Action control: from cognition to behavior.

[37] Cathébras P, Begon A, Laporte S, Bois C, Truchot D (2004). Épuisement professionnel chez les médecins généralistes. Presse Med.

[38] Texier G, Rhondali W, Morel V, Filbet M (2013). Refus de prise en charge du patient en soins palliatifs (en phase terminale) à domicile par son médecin généraliste: est-ce une réalité?. Méd Palliative.

[39] Delbrouck M (2012). Le burn-out du soignant: le syndrome d’épuisement professionnel.

[40] Fougere B. Prise en charge des patients douloureux en soins palliatifs par les médecins généralistes*.* Méd Palliative*:*Volume 11 ; issue 2 ; avril 2012p. 90–97.

[41] Loi n° 2016–87 du 2 février 2016 créant de nouveaux droits en faveur des malades et des personnes en fin de vie. https://www.legifrance.gouv.fr/eli/loi/2016/2/2/AFSX1507642L/jo/texte. Accessed 26 Feb 2020. 30512616

[42] Baudin S. Opinion des médecins généralistes niçois sur les directives anticipées de la loiLéonetti dans la prise en charge des patients en fin de vie. Méd Humaine Pathologie. *2013.<dumas-00809080>.*.

[43] Texier G (2011). Difficultés des médecins généralistes dans les prises en charge au domicile de leurs patients en soins palliatifs. [Thèse pour le doctorat en médecine], Rennes 1.

[44] Lawniczak L (2008). Le médecin généraliste et les soins palliatifs: besoins ressentis en matière de formation et attentes vis-à-vis des réseaux de soins. [Thèse de doctorat de médecine], Lille 2.

[45] Legendre J-P (2012). Prise en charge palliative à domicile par les médecins généralistes du territoire du réseau de soins palliatifs de Mayenne. [Thèse de doctorat en médecine], Angers.

[46] Dubertrand JM. L’hospitalisation à domicile: le point de vue du médecin généraliste: enquête sur le bassin cannois*.* [Thèse d’exercice, médecine]: Université Côte d’Azur; 2009..

[47] Crotet R, Jehenne B (2011). Attentes des médecins généralistes vis-à-vis de l’hospitalisation à domicile: étude descriptive et comparative auprès de deux groupes de médecins généralistes du secteur de l’HAD de Grenoble.

[48] Claris S (2007). Enquête menée auprès des médecins généralistes afin d’évaluer leur satisfaction vis-à-vis d’une structure d’hospitalisation à domicile: l’HAD 63 [Thèse d’exercice].

[49] Marrilliet A, Ruhlmann C, Laval G, Labarère J. Intérêt d’une permanence téléphonique de soins palliatifs. Enquête postale auprès des médecins généralistes isérois*.* /data/revues/16366522/v12i1/S1636652212000372/ [Internet]. 19 févr 2013 [cité 19 juill 2018]; Available from: http://www.em-consulte.com/en/article/789095.

[50] Barbaroux A, Jedat V (2020). Lifelong continuing education. Critical analysis of scientific and medical information. Management of links of interest. Exercer.

[51] Thoonsen B, Groot M, Verhagen S (2016). Timely identification of palliative patients and anticipatory care planning by GPs: practical application of tools and a training programme. BMC Palliat Care.

[52] Thoonsen B, Gerritzen SHM, Vissers KCP (2019). Training general practitioners contributes to the identification of palliative patients and to multidimensional care provision: secondary outcomes of an RCT. BMJ Support Palliat Care.

[53] *La Démographie médicale en région Provence-Alpes-Côte d’Azur. Situation en 2013.* [Internet]. [cited 23 juill 2018]. Available from: https://www.conseil-national.medecin.fr/sites/default/files/paca_2013.pdf.

[54] Chapdelaine P, Bissonnier C, Boetsch D, Matuszewski *C.* atlas de la démographie médicale en France, situation au 1er janvier 2014*.* Cons Natl Ordre Médecins.: 274.

[55] Boelen C (2011). Global Consensus On Social Accountability of Medical Schools. Sante Publique Vandoeuvre--Nancy Fr.

[56] Loi n° 2004–810 du 13 août 2004 relative à l’assurance maladie. https://www.legifrance.gouv.fr/affichTexte.do?cidTexte=JORFTEXT000000625158. Accessed 26 Feb 2020.

[57] Arrêté du 25 novembre 2011 portant approbation de l’avenant n° 3 à la convention nationale des infirmières et des infirmiers libéraux.

