# Supplementary material for: Home-based palliative care management: what are the useful resources for general practitioners? a qualitative study among GPs in France
Source: BMC Fam Pract. 2020 Oct 31;21:222. doi: 10.1186/s12875-020-01295-7 (PMC7603704; doi:10.1186/s12875-020-01295-7)
Supplement: Supplementary file 1 — Additional file 1:. Interview guide. [file 12875_2020_1295_MOESM1_ESM.docx]

**Interview Guide** (translated from French)

*"Hello, as I told you on the phone, I am writing a thesis about palliative care and end-of-life as managed by general practitioners. While gathering literature before starting my thesis, I read a lot of rather negative things letting me think that general practitioners tend to disinvest from taking care of end-of-life patients; yet, I do not think this is the case, and the goal of my thesis is to list the methods that physicians use to successfully manage these patients and the inherent difficulties they encounter when doing so. "*

1. When you first set up, what were the main problems you were confronted with when managing palliative care patients?
2. And today, does it seem simpler? What solutions did you find?
3. Any other problems? (here comes additional probing if needed)
4. We’ve broached all the problems I was thinking of; would you like to add anything? Is there a disincentive or a resource we didn’t talk about? Any comments or suggestions?

Additional question topics:

- Training
- Financing
- Work time and work load
- Relationships with independent paramedical health professionals
- Symptom management (pain and other)
- Relationships with the patient’s relatives
- Work within independent networks
- Assistance structures
